# Supplementary material for: Efficient conversion of chemical energy into mechanical work by Hsp70 chaperones
Source: eLife. 2019 Dec 17;8:e48491. doi: 10.7554/eLife.48491 (PMC7000219; doi:10.7554/eLife.48491)
Supplement: Figure 2—source data 1. [file elife-48491-fig2-data1.zip › Fig2/Figure_2_readme.pdf]

Data for figure 2

Rg_vs_G.dat = Mean Rg (first column) and free energy $\Delta\Delta G$ (second column, expressed in kcal/mol) for each realization (corresponding to the points in the main plot)

Sanchez_curve.dat = Rg (first column) and free energy (second column) used for the black curve

histogram_Free_Energy.dat = values for the histogram in the inset.
