# Supplementary material for: Efficient conversion of chemical energy into mechanical work by Hsp70 chaperones
Source: eLife. 2019 Dec 17;8:e48491. doi: 10.7554/eLife.48491 (PMC7000219; doi:10.7554/eLife.48491)
Supplement: Figure 4—source data 1. [file elife-48491-fig4-data1.zip › Fig4/Figure_4_readme.pdf]

Figure 4 data

p_n_k.dat = ratio [ATP]/[ADP] (first column) and corresponding probability (second column) for the state with n=k chaperones bound (k =0,1,2...,6).

Mean_N_equilibirum_bottom.dat = ratio [ATP]/[ADP] (first column) mean number of chaperones (second column) in the case of equilibrium (dashed black line in the bottom panel)

Mean_N_nonequilibirum_bottom.dat = ratio [ATP]/[ADP] (first column) mean number of chaperones (second column) in the case of non equilibrium (continuous black line in the bottom panel)
