# Supplementary material for: Efficient conversion of chemical energy into mechanical work by Hsp70 chaperones
Source: eLife. 2019 Dec 17;8:e48491. doi: 10.7554/eLife.48491 (PMC7000219; doi:10.7554/eLife.48491)
Supplement: Figure 5—source data 1. [file elife-48491-fig5-data1.zip › Fig5/Figure_5_readme.pdf]

Data figure 5

FRET :

FRET_EQ_k.dat = average FRET for equilibrium [ATP]/[ADP] ratio in realization k (inset, blue)
FRET_NE_k.dat = average FRET for non equilibrium [ATP]/[ADP] ratio in realization k (inset, red)

Average_FRET_Efficiency.dat = experimental values (inset, black)

Rg :

Rg_histogram_equilibrium.dat = data for the histogram of Rg in equilibrium (main, blue)
Rg_histogram_non_equilibrium.dat = data for the histogram of Rg in non equilibrium (main, red)
