# Supplementary material for: Efficient conversion of chemical energy into mechanical work by Hsp70 chaperones
Source: eLife. 2019 Dec 17;8:e48491. doi: 10.7554/eLife.48491 (PMC7000219; doi:10.7554/eLife.48491)
Supplement: Figure 6—source data 1. [file elife-48491-fig6-data1.zip › Fig6/Figure_6_readme.pdf]

Data Figure 6

Top panel

Effectivenessk.dat = for the k realization (k=1,…,30), the ratio between the free energy of swelling and the one of hydrolysis is given (second column) as a function of the [ATP]/[ADP] ratio (first column). These data have been used for the green curves in the top panel.

Bottom panel

Kd_ren.dat = [ATP]/[ADP] ratio (first column) and corresponding normalized dissociation constant for the case of single binding site (second column). These data were used for the black curve in the bottom panel.

Eff_singleBS.dat = [ATP]/[ADP] ratio (first column) and corresponding ratio between the free energy of binding and the one of hydrolysis (second column). These data were used for the purple curve in the bottom panel.
